# Effects of short-term manure nitrogen input on soil microbial community structure and diversity in a double-cropping paddy field of southern China

**DOI:** 10.1038/s41598-020-70612-y

**Published:** 2020-08-11

**Authors:** Haiming Tang, Chao Li, Xiaoping Xiao, Lihong Shi, Kaikai Cheng, Li Wen, Weiyan Li

**Affiliations:** Hunan Soil and Fertilizer Institute, Changsha, 410125 People’s Republic of China

**Keywords:** Biochemistry, Ecology, Microbiology

## Abstract

The soil physicochemical properties and soil microbial communities were affected by different fertilizer management. Fertilizer regime were closely relative to the soil texture and nutrient status in a double-cropping paddy field of southern China. However, there was limited information about the influence of different manure nitrogen (N) input on soil microbial communities in a double-cropping rice (*Oryza sativa* L.) field. Therefore, the short-term different manure N input rate management on soil bacterial and fungal diversity in a double-cropping paddy field of southern China were studied by using Illumina sequencing and quantitative real-time polymerase chain reaction technology in the present paper. The filed experiment were including 100% N of chemical fertilizer (M0), 30% N of organic manure and 70% N of chemical fertilizer (M30), 50% N of organic manure and 50% N of chemical fertilizer (M50), 100% N of organic manure (M100), and without N fertilizer input as control (CK). The results showed that diversity indices of soil microbial communities with application of organic manure and chemical N fertilizer treatments were higher than that of CK treatment. Application of organic manure and chemical N fertilizer management increase soil bacterial abundance of the phylum *Actinobacteria*, *Proteobacteria* and *Gammaproteobacteria*, and soil fungi abundance of the phylum *Basidiomycota* and *Zygomycota* were also increased. Compared with CK treatment, the value of Richness, Shannon and McIntosh indices, and taxonomic diversity were increased with M30, M50 and M100 treatments. This finding demonstrated that M30, M50 and M100 treatments modify soil bacterial and fungal diversity. Therefore, the combined application of organic manure and chemical fertilizer N management could significantly increase the abundance of profitable functional bacteria and fungi species in a double-cropping rice field of southern China.

## Introduction

Soil microorganisms play an important role in biogeochemical cycle, which were affected by different fertilizer management and was closely relative with crop growth, nutrient cycling, and sustainability of soil productivity^1^. Soil microbial activity and community structure were affected by different agriculture management measures, such as the crop types, tillage, fertilizer regime, irrigation patterns, and so on^2–4^. Soil physicochemical properties were mainly affected by different fertilizer management, and the soil microbial activities and communities were also changed under application of different fertilizer condition^5^. Maintaining the complexity and diversity of soil microorganisms is critical to sustain soil fertility, because soil microbes mediate the biogeochemical cycles of carbon (C) and nitrogen (N), as well as serve as an important reservoir for plant nutrition^6^.

Microbial communities exhibit immense diversity in terms of structure (i.e. community composition) as well as function (i.e. physiology of all species combined) that vary from place to place as well as over time^7^. In recent years, the effects of different organic and inorganic N fertilizer regime on soil microbial properties were conducted by more and more researchers. Different organic and inorganic N fertilizer treatments affect microbial communities through direct influence on soil nutrient content and chemistry properties^8^. Organic N fertilizer practices generally have positive effects on various soil properties^9^, enhancing field soil structure by increasing soil organic matter (SOM) content, and thus reducing soil erosion^10^. Therefore, the influence of different organic and inorganic N fertilizer management was provided a way for us to study the soil biological processes. Some studies indicated that different organic and inorganic N fertilizer practices was major factor in affecting soil community structure, diversity, and abundance of soil microbes, Wang et al.^11^ result indicated that organic N input management enhance the diversity and stability of soil microbial community. Organic N input management had positive effects on SOM content, soil microbial community structure and diversity, and the abundance of soil bacteria and fungi^12,13^. Previous studies found that abundance, activity and diversity of soil microbes were enhanced with the return of organic manure and crop straw to soil^8,14^. In contrast, some study indicated that application of chemical N fertilizer practices reduced soil microbial community and activity^15^. However, there is still limited information about the change of soil microbial community under different manure N input rate management condition.

Rice (*Oryza sativa* L.) is the major crop in the tropical and subtropical monsoon climate regions of Asia^16^. The early rice and late rice (double-cropping) production system is the main crop system in southern of China, and the fertilizer regime (organic fertilizer, inorganic fertilizer, and so on) is an important influence factors that maintain the quality and fertility of paddy soil^17^. And the soil properties including the soil pH, SOC content were affected by different manure N input rate management, which in turn influence the soil bacteria and fungi community structure and function^18^. However, there is little information about the influences of different manure N input rate practices on soil microbial activities and communities structure in a double-cropping paddy field of southern China. We hypothesized that the function and structure of soil bacteria and fungi communities were changed under taken different manure N input rate management’s condition. Therefore, the abundance and community structure of soil bacteria and fungi were investigated by using Illumina sequencing and quantitative real-time polymerase chain reaction (PCR) technology, respectively.

As a result, the object of this study were: (1) to analyze the effect on soil microbial community following 3-year of continuous application of different manure N input management, (2) to investigate the relationship between soil bacteria, fungi community and soil properties, and (3) to choose an proper fertilizer practice in a double-cropping paddy field of southern China.

## Materials and methods

### Sites and cropping system

The experiment was beginning in 2017. It was located in Ningxiang County (28° 07′ N, 112° 18′ E) of Hunan Province, China. At the beginning of the study, the surface soil physicochemical properties (0–20 cm) were as follows: soil organic carbon (SOC) 22.75 g kg^−1^, total N 2.24 g kg^−1^, available N 178.90 mg kg^−1^, total phosphorous (P) 0.66 g kg^−1^, available P 18.45 mg kg^−1^, total potassium (K) 14.45 g kg^−1^, and available K 69.50 mg kg^−1^, pH 6.80. The climate condition (annual mean precipitation and evapotranspiration, monthly mean temperature) of experiment region, soil type and soil texture, and crop system were described by Tang et al.^19^.

### Experimental design

The experiment was including five fertilizer treatments: (1) 100% N of chemical fertilizer (M0), (2) 30% N of organic manure and 70% N of chemical fertilizer (M30), (3) 50% N of organic manure and 50% N of chemical fertilizer (M50), (4) 100% N of organic manure (M100), and (5) without N fertilizer input as control (CK). There were three replications and each plot size was 88.0 m^2^ (10.0 m × 8.0 m). The experiment ensured all fertilizer treatments received the same amount of N, phosphorus pentoxide (P_2_O_5_), potassium oxide (K_2_O) (the total amount of N, P_2_O_5_, K_2_O include chemical fertilizer and that from organic manure) during the early and late rice growing season, respectively. The types of N, P_2_O_5_ and K_2_O were including urea, ordinary superphosphate and potassium chloride, respectively. For both early and late rice, the quantity of N were applied at the rate of 135.0 and 165.0 kg ha^−1^ (60%, 30% and 10% at basal, tillering and full heading stages), 54.0 and 45.0 kg ha^−1^ of P_2_O_5_ as superphosphate, 67.5 and 90.0 kg ha^−1^ of K_2_O as potassium chloride. All the P_2_O_5_ and K_2_O fertilizer were applied at soil tillage before rice seedling transplanting. During the early rice growth period, the quantity of organic manure added into the paddy soil for the M30, M50 and M100 treatments were 828.0, 1,380.0 and 2,760.0 kg ha^−1^, respectively. During the late rice growth period, the quantity of organic manure added into the paddy soil for the M30, M50 and M100 treatments were 1,012.5, 1687.5 and 3,375.0 kg ha^−1^, respectively. And the N, P_2_O_5_ and K_2_O content of organic manure were 48.9 g kg^−1^, 17.3 g kg^−1^, and 15.1 g kg^−1^, respectively. Before transplanting rice seedlings, organic manure was manually spread onto the soil surface and incorporated into the soil at a cultivation depth of 20 cm. Plots were irrigated at the same time as the tillage management was carried out and kept at 2 cm above soil surface during transplanting of rice seedlings. Thereafter, water depth was kept at 5 cm above the soil surface during the seedling stage, followed by drainage and drying of the field at tillering stage, and finally alternating wet/dry irrigation in the later growth stages^20^.

The early and late rice seedlings were manually transplanted to the paddy in April and July, and harvested with a combine in July and October, respectively. The variety of early rice (*Oryza sativa* L.) Xiangzaoxian24 and variety of late rice Jinyou 59 were used as the materials during experiment period. The early and late rice seedlings were transplanted with a density of 280,000 plants ha^−1^.

### Soil sampling

Soil was sampled at maturity stage of late rice in late October 2019. Soil samples were collected by randomly from six cores from each plot. After removing visible organic materials, stones and rice roots by hand, the samples were divided into two parts. The fresh soil samples were placed immediately in ice box and transported to the laboratory. One part of the fresh soil sample was passed through a 2-mm mesh sieve and then stored at room temperature until soil properties analysis (for pH, SOC, and total nitrogen), and another part was stored at − 20 °C until molecular analysis. The detail information about soil sampling was described by Tang et al.^20^.

### Soil laboratory analysis

#### Physical and chemical characteristics

The physical and chemical analyses of the soil were performed in the laboratory. Measurements of pH, SOC, total nitrogen (TN), and soil porosity were performed according to Zhao et al.^21^. Soil microbial biomass carbon (SMBC) and soil microbial biomass nitrogen (SMBN) contents were measured by the fumigation-extraction method as described in Wu et al*.*^22^.

#### DNA extraction, PCR amplification, and Illumina sequencing

The concentration and quality of the soil DNA were detected following the manufacturer’s instructions. Primers pairs F515 and R806 targeting the V3-4 region of the soil bacteria 16S rRNA gene were used for PCR^23^. And the protocol for PCR amplification of the 16S rRNA gene were performed using the method described by Wang et al.^24^. And the target DNA fragment (ITS1 region) of soil fungal was amplified by PCR reaction system with ITS1 and ITS2 as primers^25^. Each PCR product was subjected to pyrosequencing using the Illumina MiSeq platforms at Xidai Biotechnology Co., Ltd., Changsha, China. The original DNA fragments were merging the pairs of reads using FLASH software^24^. Therefore, further sequence analyses were conducted using USEARCH v5.2.32^26^.

#### Sequence analysis and population identification

The merge and operational taxonomic units (OTU) partition of the obtained sequence, and select the representative sequence of each OTU by using QIIME software. Therefore, the default parameters were used to obtain the corresponding taxonomic information of each OTU by comparing the representative sequence of each OTU with the template sequence of the corresponding database. The gene database of soil bacteria was Greenenes v13.8, and the identification of soil fungi was compared with UNITE database (https://unite.ut.ee).

### Statistical analysis

The Richness diversity, Shannon diversity and McIntosh diversity indices were calculated by using Mothur software. Meanwhile, the relationship between abundance of dominant phyla and soil physicochemical properties were measured by using redundancy analysis (RDA).

The results of every measured item were presented in mean values and standard error. The data of each treatment means were compared by using one-way analysis of variance (Anova) following standard procedures at the 5% probability level. All statistical analyses of correlations between soil properties and abundant phyla were calculated by using the SAS 9.3 software package^27^.

## Results

### Soil properties

The effects of different manure N input rate management on soil pH, soil organic carbon (SOC) content, porosity, total N, soil microbial biomass carbon (SMBC), and soil microbial biomass nitrogen (SMBN) contents in a double-cropping rice field were shown in Table 1. At maturity stage of late rice, the lowest soil pH, SOC, porosity, total N, SMBC, and SMBN contents in paddy field were observed with M0 and CK treatments, while the highest soil pH, SOC, porosity, total N, SMBC, and SMBN contents in paddy field were observed with M100 treatment. Meanwhile, the results showed that soil pH, SOC, porosity, total N, SMBC, and SMBN contents with M100 treatment were significantly higher (*p* < 0.05) than that of M0 and CK treatments in a double-cropping rice paddy field.

**Table 1:** Basic soil properties with different fertilizer treatments in paddy field at maturity stage of late rice.

Treatments	pH	SOC (g kg^−1^)	Porosity (%)	Total nitrogen (g kg^−1^)	SMBC (mg kg^−1^)	SMBN(mg kg^−1^)
M0	6.78 ± 0.19b	21.72 ± 0.68b	45.2 ± 1.2b	2.24 ± 0.06ab	426.15 ± 12.30b	46.24 ± 1.41ab
M30	6.93 ± 0.17ab	22.96 ± 0.66ab	48.6 ± 1.3ab	2.26 ± 0.05ab	444.69 ± 12.83b	46.94 ± 1.37ab
M50	7.01 ± 0.18ab	23.47 ± 0.65ab	49.5 ± 1.3ab	2.31 ± 0.06a	469.05 ± 13.54ab	47.49 ± 1.36a
M100	7.15 ± 0.18a	24.03 ± 0.62a	51.7 ± 1.6a	2.42 ± 0.07a	524.99 ± 15.15a	48.81 ± 1.33a
CK	6.77 ± 0.14b	21.30 ± 0.62b	44.3 ± 1.1c	2.23 ± 0.05b	387.34 ± 11.18c	43.09 ± 1.24b

M0, 100% N of chemical fertilizer; M30, 30% N of organic manure and 70% N of chemical fertilizer; M50, 50% N of organic manure and 50% N of chemical fertilizer; M100, 100% N of organic manure; CK, without N fertilizer input as control; SOC, soil organic carbon; SMBC, soil microbial biomass carbon; SMBN, soil microbial biomass nitrogen.

Different lowercase letters in the same column indicated significantly difference at *p* < 0.05.

The same as below.

### Soil bacterial taxonomic distribution

**Figure 1:**
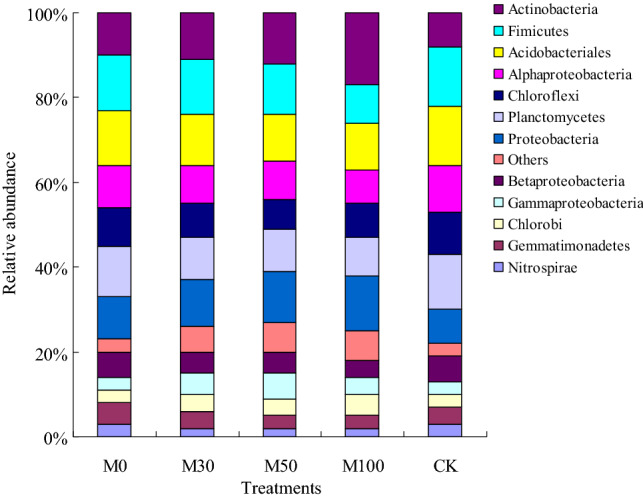
Relative abundance of the dominant bacterial phyla in all soil samples combined and in each fertilizer treatments. M0: 100% N of chemical fertilizer, M30: 30% N of organic manure and 70% N of chemical fertilizer, M50: 50% N of organic manure and 50% N of chemical fertilizer, M100: 100% N of organic manure, CK: without N fertilizer input as control. Relative abundance were based on the proportional frequencies of those DNA sequences that could be classified.

The phylum level analysis results indicated that phyla *Actinobacteria* (8–17%), *Acidobacteria* (11–14%), *Alphaproteobacteria* (8–11%), *Chloroflexi* (7–10%), *Fimicutes* (9–14%), *Proteobacteria* (8–13%) and *Planctomycetes* (9–13%) were the most abundant in the soil samples. And the phyla *Proteobacteria*, *Actinobacteria* with M30, M50 and M100 treatments were significantly higher (*p* < 0.05) than that of with M0 and CK treatments (Fig. 1). The phyla *Alphaproteobacteria*, *Acidobacteriales*, *Chloroflexi*, and *Fimicutes* with M30, M50 and M100 treatments were significantly lower (*p* < 0.05) than that of with CK treatment. The results also indicated that relative abundance of *Gammaproteobacteria* were richer higher with M30 (5%), M50 (6%) and M100 (4%) treatments soil, and the relative abundance of *Acidobacteriales* and *Chlorobi* were higher with CK (14%, 10%) treatment soil. Compared with CK treatment, the relative abundance of *Proteobacteria* and *Actinobacteria* with M100 treatment were increased 62.50% and 112.50%, respectively. Significant differences in soil bacterial composition were observed between the M30, M50, M100 and M0, CK treatments soil (Fig. 1).

### Soil fungal taxonomic distribution

**Figure 2:**
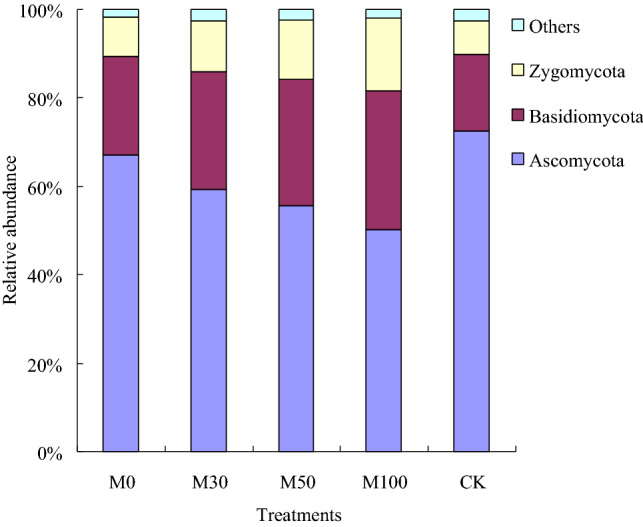
Relative abundance of the dominant fungal phyla in all soil samples combined and in each fertilizer treatments.

The results showed that phyla *Ascomycota* (50.3–72.5%), *Basidiomycota* (17.4–31.2%) and *Zygomycota* (7.5–16.6%) were the most abundant in the soil samples. And the order of phyla with different fertilizer treatments was showed *Ascomycota* > *Basidiomycota* > *Zygomycota*. And the phyla *Ascomycota* with CK (72.5%) treatment were significantly higher (*p* < 0.05) than that of with M30 (59.4%), M50 (55.6%) and M100 (50.3%) treatments (Fig. 2). The phyla *Basidiomycota* and *Zygomycota* with M30, M50 and M100 treatments were significantly higher (*p* < 0.05) than that of with M0 and CK treatments. The results indicated that relative abundance of *Basidiomycota* and *Zygomycota* were richer higher (*p* < 0.05) with M30 (26.6%, 11.3%), M50 (28.6%, 13.5%) and M100 (31.2%, 16.6%) treatments soil, and the relative abundance of *Ascomycota* were higher (*p* < 0.05) with CK (72.5%) treatment soil. Compared with CK treatment, the relative abundance of *Basidiomycota* and *Zygomycota* with M100 treatment were increased 79.31% and 121.33%, respectively.

### Soil bacterial and fungi alpha diversity

Richness, Shannon and McIntosh indices were used to reflect the richness and evenness of soil microbial community species, respectively. At soil bacterial alpha diversity, Richness indices were increased with M100 treatment compared with CK treatment, and the order of Richness indices with different fertilizer treatments was showed M100 > M50 > M30 > M0 > CK. Compared with CK treatment, the Shannon and McIntosh indices were increased with M30, M50 and M100 treatments (Table 2). Compared with CK treatment, the Richness indices, Shannon indices and McIntosh indices with M100 treatment were increased 11.25%, 43.28% and 35.39%, respectively.

**Table 2:** Soil bacterial and fungal diversity parameters with different fertilizer treatments.

Microorganism	Diversity parameters	Treatments				
		M0	M30	M50	M100	CK
Soil bacterial	Richness indices	14.57 ± 0.35b	15.05 ± 0.40ab	15.45 ± 0.41ab	15.92 ± 0.42a	14.31 ± 0.37b
	Shannon indices	4.76 ± 0.13b	5.25 ± 0.14a	5.37 ± 0.16a	5.76 ± 0.17a	4.02 ± 0.11c
	McIntosh indices	5.78 ± 0.14b	6.15 ± 0.15ab	6.43 ± 0.15a	6.58 ± 0.17a	4.86 ± 0.11c
Soil fungi	Richness indices	11.43 ± 0.35ab	11.83 ± 0.34ab	12.06 ± 0.34ab	12.35 ± 0.33a	11.15 ± 0.32b
	Shannon indices	3.76 ± 0.13b	4.02 ± 0.12ab	4.15 ± 0.12a	4.36 ± 0.11a	3.25 ± 0.09c
	McIntosh indices	4.53 ± 0.14b	4.84 ± 0.14ab	5.06 ± 0.13a	5.15 ± 0.13a	4.17 ± 0.12c

Different lowercase letters in the same line indicated significantly difference at *p* < 0.05.

The same as below.

At soil fungi alpha diversity, Richness indices were increased with M100 treatment compared with CK treatment. Compared with CK treatment, the Shannon and McIntosh indices were increased with M50 and M100 treatments (Table 2). Compared with CK treatment, the Richness indices, Shannon indices and McIntosh indices with M100 treatment were increased 10.76%, 34.15% and 23.50%, respectively. The results indicated that Richness indices, Shannon indices and McIntosh indices with application of manure N input management were higher (*p* < 0.05) than that of with chemical fertilizer and without N fertilizer input management.

### RDA of soil bacterial and fungal abundant phyla and soil characteristics

**Figure 3:**
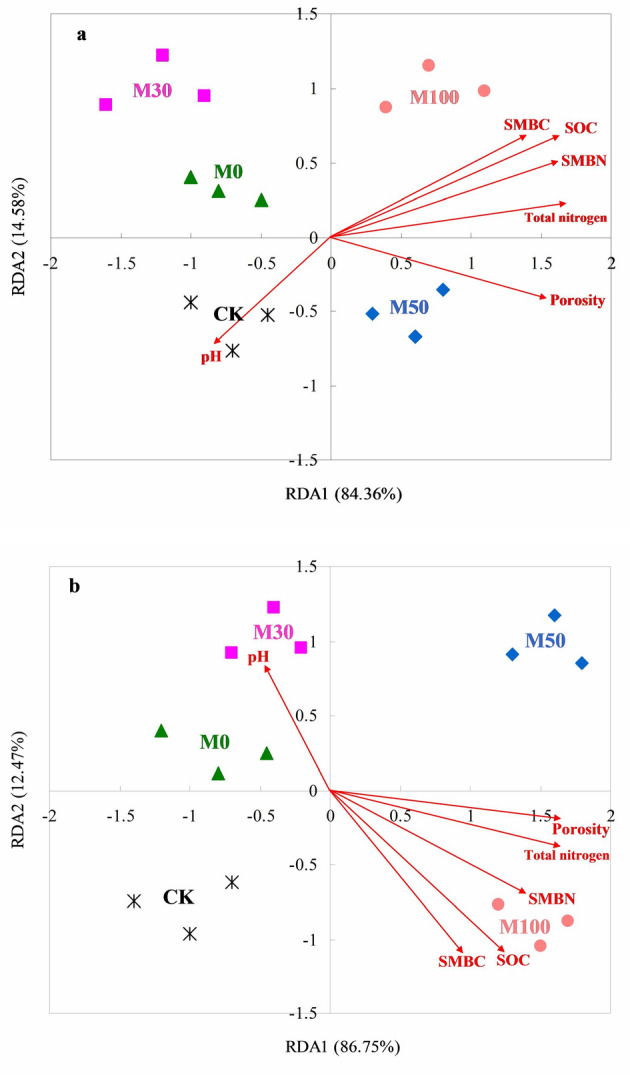
Redundancy analysis of soil bacterial (**a**) and fungal (**b**) abundant phyla and soil characteristics (arrows) of different fertilizer treatments. M0: 100% N of chemical fertilizer (triangle); M30: 30% N of organic manure and 70% N of chemical fertilizer (square); M50: 50% N of organic manure and 50% N of chemical fertilizer (prismatic); M100: 100% N of organic manure (circle); CK: without N fertilizer input as control (asterisk).

The results revealed there was a significantly correlation between soil bacterial abundant phyla and soil chemical properties (Fig. 3a). The soil properties can explain the variation (84.36% and 14.58%) in soil bacterial abundant phyla between the M0, M30, M50, M100 and CK treatments. Under different fertilizer treatments, M0 and CK treatments were separated from application of organic manure treatments (M30, M50 and M100 treatments), indicating that fertilizer treatments significantly change the characteristics of soil bacterial abundant phyla. The soil bacterial abundant phyla was significantly correlated with soil physical and chemical properties including the porosity (*r* = 0.965), SOC (*r* = 0.947), total N (*r* = 0.967), SMBC (*r* = 0.947), and SMBN (*r* = 0.913) contents. But there was negative correlation between soil bacterial abundant phyla and soil pH (*r* = −0.635).

The results indicated that soil properties can explain the variation (86.75% and 12.47%) in soil fungal abundant phyla between the M0, M30, M50, M100 and CK treatments (Fig. 3b). Under different fertilizer treatments, M0 and CK treatments were separated from application of organic manure treatments (M30, M50 and M100 treatments). The soil fungal abundant phyla was significantly correlated with the soil physical and chemical properties including the porosity (*r* = 0.961), SOC (*r* = 0.874), total N (*r* = 0.743), SMBC (*r* = 0.736), and SMBN (*r* = 0.765) contents. But there was negative correlation between soil fungal abundant phyla and soil pH (*r* = −0.608).

## Discussion

In the present study, the results showed that soil microbial community were mainly changed by different manure N input rate management, including soil structure, soil pH, SOC, total nitrogen, SMBC, and SMBN contents (Table 1), the soil total nitrogen, SOC, SMBC, and SMBN contents were increased with application of organic manure incorporation treatments compared with the without N fertilizer input treatment soil, consistent with the results of a previous studies^8,12^. Application of organic manure practices significantly increased the SOC content, the reason maybe that soil C redistribution and microbial habitat were altered under manure N input management condition^28^. Soil aggregation mediated were changed by porosity, and soil C sequestration were increased under application of organic manure N condition^29^. Based on those findings, the results were indicated that distribution of the soil bacterial and fungal communities in a double-cropping rice system were changed due to the changes in soil properties.

Compared to the chemical fertilizer and without N fertilizer input treatments (M0, CK), the organic manure incorporation treatments (M30, M50 and M100) strongly increase the Richness, Shannon and McIntosh indices in the soil samples. At the indices of soil microbial communities, the results indicated that indices of soil microbial communities were increased with application of organic manure management (Figs. 1, 2), which may be the disturb of microbiota were moderated, the soil texture and moisture content were decreased, therefore, the competitive niche and selection mechanisms among different populations were excluded. As a result, the Richness, Shannon and McIntosh indices were increased in agree with the previous studies^8,11^. This result maybe explained from the soil texture and moisture condition by using organic manure practices. The soil pore connectivity were mainly affected by soil texture and moisture, which was the important influence factor of changes in soil bacterial and fungal diversity, and was closely related to the Simpson and Shannon indices^11^. There were more nutrient and carbon source with organic manure treatments than that with chemical fertilizer and without N fertilizer input treatments, which was provide an appropriate soil environment and nutrient for soil bacteria and fungal multiplying. Therefore, this study was supports the idea that soil bacteria and fungal alpha diversity and abundance were changed with application of organic manure practices, which the soil texture, soil moisture content, and soil properties were obvious altered.

Some studies indicated that structure and diversity of soil bacterial community were changed under different fertilizer condition^1,8,11^. In the present study, the soil bacterial community was investigated by using Illumina sequencing and PCR technology. The results showed that the relative abundance of *Proteobacteria* and *Actinobacteria* with M30, M50 and M100 treatments were higher than that with M0 and CK treatments (Fig. 1), which were provide an appropriate soil environment and nutrient for *Proteobacteria* and *Actinobacteria* multiplying under application of organic manure condition. The relative abundance of *Fimicutes* with CK treatment were higher than that with M30, M50 and M100 treatments, which were mainly benefit for produce endospores under without N fertilizer input condition^21^. Jenkins et al.^30^ reported that *Proteobacteria* was a fast-growing copiotrophs under higher SOC environment, competed with soil nutrient with *Firmicutes* was the main reason for the reduced abundance of *Alphaproteobacteria* and *Betaproteobacteria* with M30, M50 and M100 treatments compared to M0 and CK treatments soil as observed in the present study. Meanwhile, the results indicated that abundance of *Alphaproteobacteria* with M0 and CK treatments soil were higher than that of the other treatments soil, which were considered as heterotrophic, nitrogen-fixing organisms^31^. These findings were significantly correlated with soil texture and soil nutrient condition change (Table 1), which was main reason for the higher relative abundance of *Alphaproteobacteria* with M0 and CK treatments soil than that of with other fertilizer treatments soil. Some study indicated that abundance of *Acidobacteriales* and *Chloroflexi* were regarded as oligotrophs^32^, and its soil textures and soil nutrient contents were the main reason for the lower abundance of *Acidobacteriales* and *Chloroflexi* with organic manure treatments soil than that with M0 and CK treatments soil. Therefore, fraction of soil texture were changed, soil aeration and nutrient contents were improved with organic manure treatments, thus the relative abundance of profitable functional bacteria species were increased.

There were significantly differences in response patterns of soil fungal community structure and diversity to different fertilizer regime. In this study, the results showed that phyla *Ascomycota*, *Basidiomycota* and *Zygomycota* were the most abundant in the soil samples, consistent with the results of previous study^33^. And the phyla *Ascomycota* with organic manure treatments were significantly lower than that of with CK treatment (Fig. 2), which were considered as the growth of *Ascomycota* was not good with application of organic manure practices. The main reason maybe that it was not benefit for the growth and reproduction of *Ascomycota* under experiment soil condition. On the other hand, the application of organic fertilizer contains a lot of nitrogen, phosphorus and potassium nutrients, and the excessive application of organic manure fertilizer in the soil will promote the pathogenic characteristics of fungi^34^, leading to the inhibition of *Ascomycota* growth. Compared with M0 and CK treatments, the phyla *Basidiomycota* and *Zygomycota* were increased with application of organic manure treatments (M30, M50 and M100 treatments), which was provide nutrient and appropriate soil environment for *Basidiomycota* and *Zygomycota* multiplying. The reason may be that application of organic manure treatments can increase the SOC content, improve the ventilation condition of soil, and create favorable soil condition for *Basidiomycota* and *Zygomycota* growth. However, the application of chemical fertilizer treatment were cause the deterioration of soil physical and chemical properties and soil hardening, which was not benefit for the growth and reproduction of *Basidiomycota* and *Zygomycota*.

In the present study, the redundancy analysis results showed that soil bacterial community structure was closely related to soil porosity, SOC, total nitrogen, SMBC, and SMBN contents, and soil fungal community structure was closely related to soil porosity and SOC content. However, there were not obvious effects of soil pH on soil bacteria and fungi community structure (Fig. 3). Many studies have shown that soil microbial growth was affected by soil moisture and aeration condition^6,22^. Soil microbial biomass carbon content was also an important parameter reflecting the turnover of soil organic manure^35^, which was influence on soil bacterial and fungal community structure. Meanwhile, some studies also indicated that soil bacterial and fungal community structure were affected by soil organic matter and total nitrogen contents^12,13^. In this study, the soil fertility were improved with application of organic manure practices, and the soil microorganisms growth and reproduction, carbon/nitrogen content of soil microbial biomass were also increased^36^, and soil microbial community structure were changed. Some studies showed that soil pH has a profound impact on soil bacterial and fungal community structure^34,37^, while our research results indicated that there were not obvious effects of soil pH on soil bacterial and fungal community structure, the reason maybe that change of soil pH was not sufficient impact on soil microbial community (Table 1). Therefore, the soil microbial function was an important indicator of soil quality, which was influenced by different fertilizer management. And the mechanism of soil microbial function responds to different manure N input fertilizer management needs further study.

## Conclusions

In this study, the results indicated that soil physical and chemical properties were significantly changed under 3-year fertilizer treatments condition, and the soil bacterial and fungal diversities and community compositions were also modified in a double-cropping paddy field of southern China. And the higher value of Richness, Shannon and McIntosh indices were found in organic manure and chemical fertilizer soil, and more diversification soil bacterial community were observed in combined application of organic manure with chemical fertilizer soil, which had higher rich functional microorganisms (including *Actinobacteria*, *Proteobacteria* and *Gammaproteobacteria*), and more diversification soil fungi community were observed in combined application of organic manure with chemical fertilizer soil, which had higher rich functional microorganisms (including *Basidiomycota* and *Zygomycota*). Most importantly, the results demonstrated that fraction of soil physical and chemical properties (porosity, SOC, total nitrogen, SMBC, and SMBN contents) were improved under combined application of organic manure with chemical fertilizer condition, leading to changes of the soil bacterial and fungi community. Therefore, it was found that combined application of organic manure with chemical fertilizer is feasible management contribute to increase soil bacterial and fungi community and to build stable soil environment in a double-cropping paddy field of southern China.

## References

[1] Börjesson G, Menichetti L, Kirchmann H, Kätterer T (2012). Soil microbial community structure affected by 53 years of nitrogen fertilisation and different organic amendments. Biol. Fertil. Soils.

[2] Cui J (2020). Carbon and nitrogen recycling from microbial necromass to cope with C: N stoichiometric imbalance by priming. Soil Biol. Biochem..

[3] Xiao D, Xiao SS, Ye YS, Zhang W, He XY, Wang KL (2019). Microbial biomass, metabolic functional diversity, and activity are affected differently by tillage disturbance and maize planting in a typical karst calcareous soil. J. Soil. Sediment..

[4] Dangi S, Gao S, Duan YH, Wang D (2020). Soil microbial community structure affected by biochar and fertilizer sources. Appl. Soil Ecol..

[5] Geisseler D, Scow KM (2014). Long-term effects of mineral fertilizers on soil microorganisms: a review. Soil Biol. Biochem..

[6] Trivedi P (2017). Soil aggregation and associated microbial communities modify the impact of agricultural management on carbon content. Environ. Microbiol..

[7] Jia X, Li XD, Zhao YH, Wang L, Zhang CY (2019). Soil microbial community structure in the rhizosphere of *Robinia pseudoacacia* L. seedlings exposed to elevated air temperature and cadmium-contaminated soils for 4 years. Sci. Total Environ..

[8] Zhong W (2010). The effects of mineral fertilizer and organic manure on soil microbial community and diversity. Plant Soil.

[9] Hartmann M, Frey B, Mayer J, Maeder P, Widmer F (2015). Distinct soil microbial diversity under long-term organic and conventional farming. ISME J..

[10] Francioli D, Schulz E, Lentendu G, Wubet T, Buscot F, Reitz T (2016). Mineral versus organic amendments: microbial community structure, activity and abundance of agriculturally relevant microbes are driven by long-term fertilization strategies. Front. Microbiol..

[11] Wang Y, Li CY, Tu C, Hoyt GD, DeForest JL, Hu SJ (2017). Long-term no-tillage and organic input management enhanced the diversity and stability of soil microbial community. Sci. Total Environ..

[12] Treonis AM, Austin EE, Buyer JS, Maul JE, Spicer L, Zasada IA (2010). Effects of organic amendment and tillage on soil microorganisms and microfauna. Appl. Soil Ecol..

[13] Forge TA, Hogue EJ, Neilsen G, Neilsen D (2008). Organic mulches alter nematode communities, root growth and fluxes of phosphorus in the root zone of apple. Appl. Soil Ecol..

[14] Ahn J, Lee SA, Kim JM, Kim M, Song J, Weon H (2016). Dynamics of bacterial communities in rice field soils as affected by different long-term fertilization practices. J. Microbiol..

[15] Zhu XC, Sun LY, Song FB, Liu SQ, Liu FL, Li XN (2018). Soil microbial community and activity are affected by integrated agricultural practices in China. Eur. J. Soil Sci..

[16] Yang XY, Ren WD, Sun BH, Zhang SL (2012). Effects of contrasting soil management regimes on total and labile soil organic carbon fractions in a loess soil in China. Geoderma.

[17] Chen ZD, Ti FS, Chen F (2017). Soil aggregates response to tillage and residue management in a double paddy rice soil of the southern China. Nutr. Cycl. Agroecosyst..

[18] Wei X, Zhu Z, Wei L, Wu J, Ge T (2019). Biogeochemical cycles of key elements in the paddy-rice rhizosphere: microbial mechanisms and coupling processes. Rhizosphere.

[19] Tang HM (2019). Effects of different soil tillage systems on soil carbon management index under double-cropping rice field in southern China. Agron. J..

[20] Tang HM (2020). Organic manure managements increases soil microbial community structure and diversity in double-cropping paddy field of southern China. Agric. Ecosyst. Environ..

[21] Zhao J (2014). Pyrosequencing reveals contrasting soil bacterial diversity and community structure of two main winter wheat cropping systems in China. Microb. Ecol..

[22] Wu J, Joergensen RG, Pommerening B, Chaussod R, Brookes PC (1990). Measurement of soil microbial biomass by fumigation–extraction-an automated procedure. Soil Biol. Biochem..

[23] Peiffer JA (2013). Diversity and heritability of the maize rhizosphere microbiome under field conditions. PNAS.

[24] Wang ZT, Liu L, Chen Q, Wen XX, Liao YC (2016). Conservation tillage increases soil bacterial diversity in the dryland of northern China. Agron. Sustain. Dev..

[25] Bazzicalupo AL, Bálint M, Schmitt I (2013). Comparison of ITS1 and ITS2 rDNA in 454 sequencing of hyper diverse fungal communities. Fungal Ecol..

[26] Edgar RC (2010). Search and clustering orders of magnitude faster than BLAST. Bioinformatics.

[27] SAS (2008). SAS Software of the SAS System for Windows.

[28] Blanco-Canqui H, Ferguson RB, Shapiro CA, Drijber RA, Walters DT (2014). Does inorganic nitrogen fertilization improve soil aggregation? Insights from two long-term tillage experiments. J. Environ. Qual..

[29] Neumann D, Heuer A, Hemkemeyer M, Martens R, Tebbe CC (2013). Response of microbial communities to long-term fertilization depends on their microhabitat. FEMS Microbiol. Ecol..

[30] Jenkins SN (2010). Taxon specific responses of soil bacteria to the addition of low level C inputs. Soil Biol. Biochem..

[31] Li H (2014). Soil bacterial communities of different natural forest types in northeast China. Plant Soil.

[32] Pascault N (2013). Stimulation of different functional groups of bacteria by various plant residues as a driver of soil priming effect. Ecosystems.

[33] He JZ, Zheng Y, Chen CR, He YQ, Zhang LM (2008). Microbial composition and diversity of an upland red soil under long-term fertilization treatments as revealed by culture-dependent and culture-independent approaches. J. Soil. Sediment..

[34] Paungfoo-lonhienne C (2015). Nitrogen fertilizer dose alters fungal communities in sugarcane soil and rhizosphere. Sci. Rep..

[35] Huang XM, Liu SR, Wang H, Hu ZD, Li ZG, You YM (2014). Changes of soil microbial biomass carbon and community composition through mixing nitrogen-fixing species with *Eucalyptus urophylla* in subtropical China. Soil Biol. Biochem..

[36] Desantis TZ (2006). Greengenes, a chimera-checked 16S rRNA gene database and workbench compatible with ARB. Appl. Environ. Microb..

[37] Iovieno P, Alfani A, Bååth E (2010). Soil microbial community structure and biomass as affected by *Pinus pinea *plantation in two mediterranean areas. Appl. Soil Ecol..

